# “Is a game really a reason for people to die?” Sentiment and thematic analysis of Twitter-based discourse on Indonesia soccer stampede

**DOI:** 10.3934/publichealth.2023050

**Published:** 2023-09-05

**Authors:** Otobo I. Ujah, Chukwuemeka E Ogbu, Russell S. Kirby

**Affiliations:** Chiles Center, College of Public Health, University of South Florida, 33612 Tampa Florida, USA

**Keywords:** stampede, mass gatherings, twitter, natural language processing, disaster preparedness

## Abstract

This study examined discourses related to an Indonesian soccer stadium stampede on 1st October 2022 using comments posted on Twitter. We conducted a lexicon-based sentiment analysis to identify the sentiments and emotions expressed in tweets and performed structural topic modeling to identify latent themes in the discourse. The majority of tweets (87.8%) expressed negative sentiments, while 8.2% and 4.0% of tweets expressed positive and neutral sentiments, respectively. The most common emotion expressed was fear (29.3%), followed by sadness and anger. Of the 19 themes identified, “Deaths and mortality” was the most prominent (15.1%), followed by “family impact”. The negative stampede discourse was related to public concerns such as “vigil” and “calls for bans and suspension,” while positive discourse focused more on the impact of the stampede. Public health institutions can leverage the volume and rapidity of social media to improve disaster prevention strategies.

## 1. Introduction

The occurrence of human stampedes is a significant health-related hazard in mass gathering events. These incidents involve a group of people running in the same direction, leading to injuries and deaths due to trauma [1]. As a result, mass gathering events have become a public health concern, often associated with significant mass casualty incidents [2],[3]. According to the World Health Organization (WHO), mass gathering events involve the aggregation of individuals at a specific place and time for a particular purpose, whether planned or spontaneous. Such events tend to overwhelm the preparation and allocation of resources by the host community or country [4]. As the global population continues to grow, and migration increases, mass gatherings have become more frequent, heightening the risk of such hazards [5]. Apart from stampedes, mass gatherings are associated with various other health hazards, including water and sanitation-related diseases, non-communicable diseases, exacerbation of pre-existing health conditions, transmission of communicable diseases, mental health and psychological disorders, thermal disorders such as dehydration, accidents, trauma, crush injuries, terrorist attacks, and alcohol and substance abuse [2],[3]. While many of these hazards are challenging to predict, implementing preventive measures can mitigate their impact.

The mechanisms underlying stampedes present a topic for ongoing research and understanding [6],[7]. Presently, it is known that these incidents typically stem from a triggering event that evokes an emotional response from the public, leading to a crowd crush, resulting in injuries, fatalities, and disabilities [8]. While stampedes are often associated with events such as sports gatherings, music festivals, and political rallies, they can occur in a variety of contexts [6]. Interestingly, the risk and fatality rates of stampedes tend to be higher in less developed countries compared to developed ones [9],[10]. A study [6] examining data between 1980 and 2007 revealed 215 stampedes worldwide, resulting in 14078 injuries and 7069 deaths. Notably, 27.9% of these incidents occurred in South Asia, while 25.1% were in Africa. Despite this compelling evidence, empirical research into the nature of stampedes remains insufficient [5]. There is still much to explore and comprehend to enhance our knowledge and to further implement effective preventive measures.

On Saturday, October 1, 2022, a tragic stampede occurred at a soccer stadium in Indonesia, resulting in a reported devastating loss of at least 125 lives, alongside numerous individuals being severely injured [11]. This unfortunate event, along with the recurrence of stampedes during sporting events, particularly in developing countries, emphasizes the urgent need for significant attention from the public health sector. In recent times, the widespread availability and extensive usage of social media platforms have emerged as influential factors shaping and reflecting people's knowledge, attitudes, and perceptions, as well as influencing communication patterns and information exchange [12]. Leveraging social media data has become a valuable tool for researchers to gain insights into the scale of disasters, providing valuable information for the development of prevention and mitigation strategies. Social media offers advantages such as real-time updates, vast amounts of collected data, and easy accessibility and searchability of information [13],[14]. Empirical studies increasingly acknowledge the role of big data from social media in rapidly assessing disaster responses and evaluating damage [15]. Despite numerous studies utilizing natural language processing techniques to gain insights into natural disasters through social media data [16]–[23], there remains a noticeable scarcity of research examining public perceptions and attitudes towards stampedes using these platforms [14]. Filling this research gap is crucial to understanding public sentiments and reactions to such incidents, ultimately helping to improve disaster preparedness and response strategies.

Stampedes, epidemics, and natural disasters are all emergencies that can potentially lead to loss of life. However, several distinctions set stampedes apart from epidemics and natural disasters. Stampedes tend to occur suddenly and are often brought under control relatively quickly, as compared to the gradual development of epidemics and natural disasters [24]. Additionally, the contagion effects of stampedes are immediate and confined to the incident's immediate vicinity, whereas epidemics and natural disasters can spread geographically, thereby affecting a broader population and potentially leading to long-term emotional and behavioral changes.

Despite the increasing volume of textual data available, leveraging big data from social media for qualitative research presents challenges. Issues such as premature sampling, early text selection, and difficulties in systematically and reproducibly processing and analyzing large volumes of unstructured texts can arise [25],[26]. However, advancements in unsupervised machine learning techniques and computational text analytics, including sentiment analysis and topic modeling, offer potential solutions to overcome these challenges [27],[28]. By utilizing natural language processing (NLP) techniques, researchers can identify public perceptions, attitudes, and detect latent topics embedded within big data [26],[28]. These approaches open up new avenues to gain valuable insights and understanding from the vast amount of social media data available for research.

Social media data has proven to be a valuable resource for studying various disasters in previous research. For instance, one study focused on crowd detection following the 2014 Shanghai New Year's Eve stampede, utilizing data from Weibo, a Chinese microblogging website [20]. Additionally, researchers have leveraged social media big data to study hurricanes and other natural hazards [15]–[19]. Notably, a recent study by Amoudi et al. [14] emphasized the crucial role of social media in tracking stampedes and mitigating risks through the analysis of social media data. These studies, along with other disaster-related research [15],[29]–[31], collectively demonstrate the invaluable contribution of social media data in advancing the field of disaster research. The utilization of such data opens up new possibilities for gaining insights into disaster events, understanding public sentiments and reactions, and developing strategies for disaster preparedness and response.

Our study employed NLP techniques to analyze the public discourse surrounding the Indonesian soccer stadium stampede, using textual data from a prominent social media platform. Our primary objective was to identify critical aspects of the disaster that could inform the development of effective public health strategies for disaster preparedness and responses. To achieve this goal, we sought to answer the following research questions:

RQ1: What sentiments have been expressed on social media in response to the Indonesian soccer stadium stampede?

RQ2: What underlying themes emerged from the social media discourse following the Indonesian soccer stadium stampede?

## 2. Materials and Methods

### 2.1. Data Source and Collection

The approach employed in this study involved the following steps: data collection, data cleaning and preprocessing and data analyses. All steps were conducted using R (version 4.2.1). Access to Tweets for this study was obtained after consenting to the Twitter user agreement. Data for this study were obtained from Twitter, a popular microblogging platform that has approximately more than 300 million active users monthly [20]. Twitter allows users to post user-generated content containing up to 280 characters [14]. We used Twitter's official Application Programming Interface (API) and the “*search*_*tweets*()” function contained in the “*twitteR*” package to retrieve a random sample of publicly available English-language tweets related to the Indonesian soccer stampede, which were posted from 1st–5th October 2022. However, retweets and non-English language tweets were excluded.

### 2.2. Ethics approval of research

This study did not require institutional review board approval as we used publicly available de-identified data.

### 2.3. Data cleaning and preprocessing

We performed data cleaning and preprocessing techniques to remove noise, which are usually present in unstructured texts such as in social media data. This involved the replacement of contractions and the removal of special characters (“&”, “@”, “$”, “#”), numbers, punctuations, username mentions, non-American Standard Code for Information Interchange (ASCII) characters from strings, weblinks, unicode, whitespaces, emojis, sentence breaks, duplicate tweets and stop words. Additionally, texts were converted to lower case and only tweets containing more than four words were retained. All words were tokenized into single words. To maintain the anonymity of Twitter users, we replaced users' account names with ‘@username’ when presenting phrases and quotes.

### 2.4. Data analysis

#### 2.4.1. Sentiment analysis

A sentiment analysis examines opinions, sentiments and emotions contained in the unstructured textural data [32]. The classification of texts into sentiments can be performed using either lexical methods, machine learning algorithms or a combination of both [32]. Each of these approaches can be performed at either the aspect, sentence or document level. In this study, we employed the lexicon-based approach to the sentiment analysis using the “*syuzhet*” library in R to identify prevalent sentiments and emotions expressed in the corpus. In terms of sentiment polarity, the *nrc* algorithm was applied to an emotion dictionary to score each tweet into positive, neutral and negative sentiments. On the other hand, an emotion analysis was performed based on the National Research Council (NRC) Word-Emotion Association Lexicon dictionary. The algorithm of the NRC lexicon is comprised of 14182 words and applies an emotion dictionary to categorize tweets based on Plutchik's wheel of emotions into eight emotional classifications (anger, anticipation, disgust, fear, joy, sadness, surprise and trust) [33],[34]. To do so, we used the *get_sentiment()* function in R. This function calculated sentiment scores for each word in the text using the NRC Word-Emotion Association Lexicon. Thereafter, the sum of the sentiment scores for each emotion was computed.

#### 2.4.2. Topic modeling

The size and unstructured nature of social media data makes manual classification of its content or functions into themes a challenging task [18]. Fortunately, this can be overcome using computational approaches. One such approach is the use of topic modeling. Topic modeling is an unsupervised machine learning approach based on Bayes' hierarchical model, which is used to identify latent topics within textual data by analyzing patterns among words and groups documents with similar patterns [18],[35]. While different approaches to topic modeling exist (e.g., Latent Dirichlet Analysis), in this study, we employed structural topic modeling (STM) with spectral initialization to identify latent themes in the Twitter discourse of the Indonesian soccer stampede. STM extends the framework of LDA and has the advantage of incorporating metadata as model covariates, which could provide further insight into how topic prevalence varies by these covariates [35]. To perform the STM, we used the R package “*stm*” [36] to (i) estimate the model with the optimal number of topics, (ii) visualize, label and interpret topics and (iii) cluster topics.

Given that the optimal number of topics (*K*) were unknown *a priori*, we estimated the number of topics using the “*searchK*()” to fit models beginning with 5 topics to a maximum of 50 topics, in increments of 5. Afterwards, each model was evaluated to select the best potential candidate topic model by examining the semantic coherence-exclusivity plot. To select the best model, we iteratively compared metrics from the model comprised of the semantic coherence, exclusivity, residuals and held-out likelihood. We selected the model which possessed a high semantic coherence, high held-out likelihood, high exclusivity, and low residual (i.e., high external validity and most semantically coherent and distinct topics) [35],[37].

After selecting the model with an optimal number of topics, we further examined each topic for the commonly occurring words based on the highest probability, frequency and exclusivity (FREX), score, and lift metrics and then subjectively assigned labels and interpreted each topic. The highest probability metric refers to words with the highest frequency of occurrence within each topic. On the other hand, FREX estimates the weights of words based on their frequency in a topic and by their degree of exclusiveness to that particular topic. Lift assigns higher weights to words which occur less frequently in other topics. The score is a weighting of words based on the logarithm of the frequency of a word in a specific topic divided by the logarithm of the frequency of the word in other topics.

In addition to selecting the best topic model, we examined the variation of topic prevalence by sentiment polarity and performed a correlation network analysis to group topics into distinct clusters to examine the correlation between the topics. Clustering categorizes topics into several groups in such a way that the intra-cluster similarity is greater than inter-cluster similarity. By doing so, input documents which share similar traits are segregated and assigned to clusters to provide an improved contextual comprehension.

## 3. Results

A total of 46340 original tweets in the English language were retrieved, originating from 10391 unique accounts. After data preprocessing and the removal of duplicates, 16054 tweets posted by 9998 accounts remained for analysis. Among the accounts included in the analysis, the majority (n = 8066, 80.7%) only sent one tweet. On average, each account contributed 1.6 tweets (SD = 2.8). The number of followers for each account ranged from 0 to 6402124, with a median of 995. The collected tweets received a total of 279422 retweets, with an average of 17.4 retweets per tweet (SD = 306.9). Additionally, the tweets garnered a total of 962807 favorites, averaging 60 favorites per tweet (SD = 1005.7) from other users. Furthermore, 17.5% of the accounts included in the analysis were verified. The most commonly used platform for posting tweets was Twitter for Android (21.9%), followed by the Twitter Web App (19.6%) and Twitter for iPhone (16.4%). Out of the 16054 tweets analyzed, they contained a total of 301746 words and 767321 characters.

### 3.1. Sentiment analysis

There was generally a negative sentiment valence towards the soccer stampede, with a mean sentiment value of -1.21 (SD = 1.04). The majority of tweets (87.8%, n = 14078) were classified as expressing negative sentiments towards the soccer stampede, while 8.2% (n = 1315) expressed a positive sentiment and 4.0% (n = 647) expressed a neutral sentiment. Of the emotions expressed in the corpus of tweets, fear was the most common emotion (29.3%) expressed in the tweets, with topics including the fear about the possible rise in death toll, dangers associated with playing football in Indonesia and the firing of tear gas by the police.

[Examples: “*I just saw about the stadium stampede in Indonesia and once again im reminding yall that human stampedes are so fucking unbelievably dangerous and thats exactly why its not cool or funny to wave around a toy gun or shout fire or bomb cos you think it'd be hilarious, people die fr*”

and

“*The stadium turned into a smoke-filled battleground when police fired tear gas*.”].

Sadness was the second most common emotion (19.1%) expressed and tweets included themes of rowdiness associated with football and disappointment with the stampede.

[Example: “*Deepest sympathies and condolences to the families of victims of #Malang city stampede. The EU stands with Indonesia ID in this moment of great sadness* @username.”]

and

“*Watching the football situation that happened in indonesia on tiktok fyp with sang dewi song make me wanna cry so bad* 
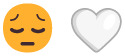
”.

Anger was the next most prevalent emotion expressed in tweets and accounted for 15.9%.

[Examples: “*Their team lost, so they got “angry”, stormed the field, and now 127 people are dead*.”

and

“*I'm both devastated and furious to hear that at least 174 people have died after teargas was fired at a football match in Indonesia. No one should die of “chaos, overcrowding, trampling and suffocation*”]

The least common emotions found in tweets were joy (2.6%), surprise (5.5%) anticipation (6.6%).

### 3.2. Topic modeling

In determining the model with the optimal number of topics, the best potential candidate topic models were estimated to between 15 and 20 topic models. To obtain a more parsimonious model, we reevaluated the topic models by specifying the number of topics starting with 10 and incrementally added one topic at a time, to a maximum of 25. After examining the exclusivity-semantic coherence plot and the output from the model diagnostic metrics, the model with 19 topics was selected. Next, we examined the prevalence of topics and compared the variation across positive and negative stampede-related discourses. Table 1 illustrates the resulting 19-topic structure characterizing the soccer stampede discourse.

The most prominent topic was “deaths and mortality” (Topic 10), which referred to deaths occurring as a result of the stampede. A common narrative within topic “*I'm sorry to hear about the tragedy of football in Malang, Indonesia on October 1st. I couldn't help thinking why the police threw tear gas at the supporters who didn't come down to the field until it killed many people. You cops are murderers. I was so sad to hear this news*. The second and third prominent topics focused on how families were impacted by the stampede (topic 11) and expression of condolences (topic 15), respectively. Less prominent topics within the stampede-related discourse were calls for ban and suspension of the league (topic 16), imposition of sanctions (topic 13), and the sack of the Indonesian police chief (topic 6).

**Figure 1.**
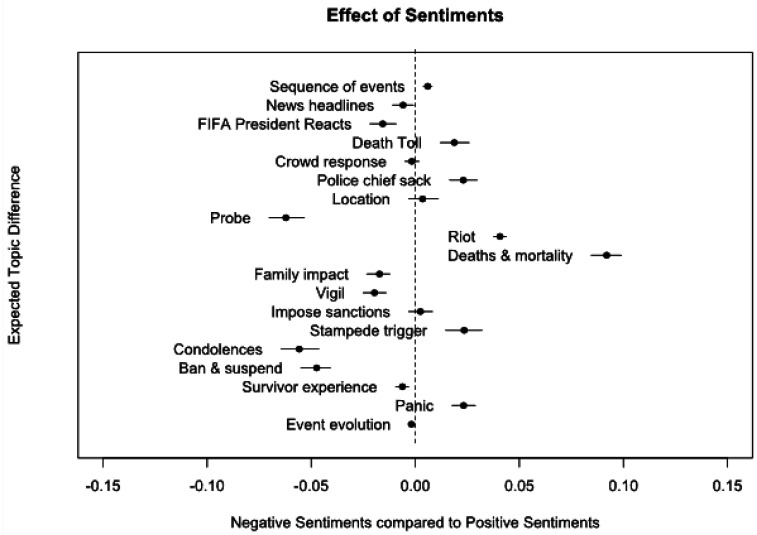
Analysis of changes in the prevalence of topics based on negative and positive sentiments.

We compared the estimated topic prevalence across tweets by sentiment polarity. Figure 1 shows the topic prevalence contrast between positive and negative sentiments. We observed “Riot” and “Death and mortality” to have a strong prevalence in the positive sentiments. Other topics which had a significant prevalence in the positive sentiment included “death toll”, “sack of police chief”, “stampede trigger” and “panic”. The topics “probe”, “condolence”, and “ban and suspension” had a strong prevalence in the negative sentiment.

**Figure 2.**
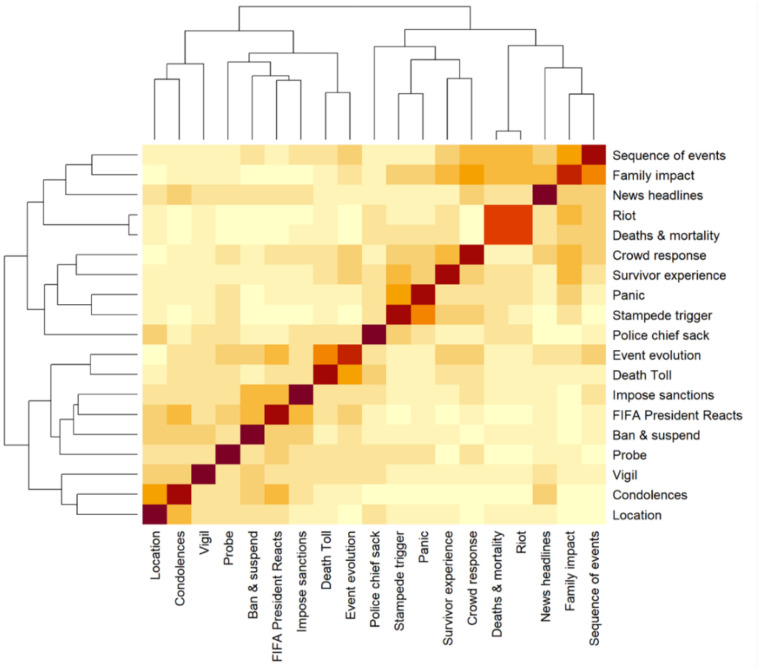
Correlation matrix and hierarchical clustering based on the distribution of topics in stampede-related Tweets.

Figure 2 presents a heatmap showing the correlations among topics listed in table 1. We observed that panic was positively correlated with the stampede trigger, while the imposition of sanctions had a substantial negative correlation with family impact.

**Table 1.** Topic modeling analysis of Tweets.

**Topic**	**Topic label**	**Proportion (%)**	**Top-10 words**	**FREX**	**Lift**
T10	Deaths & Mortality	15.1	peopl, match, kill, game, polic, break, dozen, tonight, grow, washington	kill, peopl, break, match, grow, dozen, game	grow, kill, washington, break, peopl, dozen, tonight
T11	Family Impact	8.7	dead, fan, match, player, arm, cop, lose, wife, daughter, teenag	arm, dead, player, fan, wife, daughter, cop	arm, daughter, wife, player, teenag, dead, cop
T15	Condolences	8.1	famili, victim, happen, live, condol, lost, sad, incid, game, prayer	condol, friend, peac, love, happen, rip, live	friend, heartfelt, passion, rip, condol, deepest, extend
T14	Stampede Trigger	7.6	tear, gas, polic, fire, pitch, trigger, invad, fan, control, angri	trigger, invad, pitch, tear, respond, gas, quell	shatter, invad, respond, watchdog, trigger, quell, invas
T9	Riot	5.9	iot, injur, report, video, leav, cnn, sky, unrest, footballmatch, photo	riot, cnn, report, injur, sky, video, policesay	policesay, cnn, sky, footballmatch, riot, report, video
T3	FIFA President reacts	5.8	tragedi, sport, presid, fifa, histori, day, dark, hope, widodo, weekend	presid, dark, widodo, tragedi, joko, infantino, comprehens	comprehens, dark, expos, fix, gianni, infantino, joko
T7	Location	5.3	malang, kanjuruhan, arema, event, persebaya, clash, broke, affect, sadden, surabay	persebaya, arema, surabaya, sadden, kanjuruhan, malang, deepli	impun, surabaya, corner, persebaya, tactic, arema, hide
T4	Death Toll	5.2	death, toll, children, offici, rise, mourn, result, revis, mass, relat	toll, revis, rise, relat, reach, jump, offici	jump, ministri, toll, earlier, reach, revis, stadiumstamped
T16	Ban & Suspend	4.6	leagu, ban, support, suspend, erupt, countri, human, intern, associ, play	leagu, suspend, play, ban, fuck, bet, danger	hooligan, institut, play, activ, asap, bet, fuck
T13	Impose Sanctions	4.5	disast, world, worst, deadliest, fifa, cup, explain, pssi, host, sanction	worst, cup, world, sanction, react, disast, pressur	asham, sever, statut, cup, worst, amateur, pressur
T6	Police chief sack	4.2	olic, java, east, chief, offic, sunday, provinc, local, support, remov	chief, east, provinc, java, remov, local, offic	chief, law, provinc, remov, sack, east, capac
T8	Probe	4.2	time, investig, team, york, nyt, secur, probe, set, includ, independ	york, nyt, independ, lee, investig, muktita, sui	form, ian, lee, role, surviv, austin, brief
T18	Panic	4.0	polic, exit, trampl, caus, panic, left, gate, fire, field, suffoc	panic, escap, caus, rush, panick, soccermatch, exit	chaotic, escap, vermaut, fled, panick, disastr, drive
T17	Survivor experiences	3.7	die, chao, due, hospit, lock, contribut, horror, wit, recount, blame	lock, contribut, horror, recount, die, survivor, wit	contribut, door, lock, recount, survivor, horror, delay
T5	Crowd response	3.3	crush, crowd, respons, mount, anger, justic, punish, call, cri, left	respons, justic, crush, mount, cri, crowd, punish	cri, justic, perpetr, respons, demand, mount, move
T2	News headlines	3.2	news, bbc, stori, read, top, terribl, score, horribl, guardian, come	news, bbc, read, articl, score, headlin, quot	headlin, quot, bbc, articl, news, found, read
T12	Vigil	2.6	saturday, night, hundr, victim, silenc, minut, octob, held, vigil, hurt	silenc, minut, octob, vigil, memori, night, kick	: imag, tribut, uefa, candlelight, kick, memori, minut
T1	Event evolution	2.3	violenc, watch, author, unfold, reuter, post, inindonesia, stadiumcrush, footballstamped, fear	violenc, inindonesia, stadiumcrush, reuter, footballstamped, timelin, answer	answer, footballstamped, inindonesia, stadiumcrush, timelin, rivalri, reuter
T19	Sequence of events	1.8	match, death, occur, led, troubl, game, stand, polic, peopl, saturday	occur, match, troubl, led, death, stand, game	troubl, led, occur, death, stand, match, game

## 4. Discussion

Given the unpredictability and increasing likelihood of disasters occurring during mass gathering events, it is crucial to comprehend the public's reactions to such incidents. The extensive amount of communication and rapid exchange of information on social media platforms, particularly Twitter, offers a valuable opportunity to gain insights into early conversations and discourse surrounding such phenomena, including disasters. In this study, we utilized advanced machine learning techniques to analyze Twitter data, aiming to understand the public's response to the soccer stadium stampede and to uncover underlying themes within the discourse. The findings from this study serve as an initial exploratory investigation, providing preliminary evidence upon which more comprehensive studies can be based.

Our findings revealed that in the days following the Indonesian soccer stadium stampede, public perceptions were predominantly negative. Emotions expressed in tweets were characterized by fear, anger, and sadness. Through a thematic analysis, we identified 19 themes within the Twitter discourse, focusing on the circumstances surrounding the stampede, the Indonesian government's response, and the crowd reactions. The majority of these themes were associated with negative sentiments. The thematic analysis was further supported by examining the correlation among the topics. Overall, while it is not surprising that the soccer stampede was largely negatively perceived by the public, a notable observation from our analysis was the limited discussion of prehospital medical interventions in the Twitter discourse of the disaster. This finding is consistent with a previous study [20] that utilized Twitter data to assess the Shanghai 2014 stampede. The themes identified through topic modeling did not indicate that an emergency medical response was a prominent topic of discussion.

Regardless of their purpose, an essential aspect of mass gathering events is the prevention and management of health hazards. When it comes to events such as soccer matches, which attract a large number of spectators and participants spread across various sections of the stadium, planning and mitigation strategies are often overwhelmed, inefficient, and uncoordinated, especially in the event of stampedes [38]. Previous studies have highlighted differences in the attitudes and behaviors of football fans and spectators compared to those of other sports, indicating higher tendencies for aggression among football fans compared to rugby fans [39],[40]. Such a significant finding suggests that medical care planning and responses for mass gathering events should be tailored according to the specific type of event. A key aspect of this is the need for a comprehensive risk assessment, which should be conducted prior to mass gathering events to determine whether the risk of disaster is high or low. Factors such as the timing of the event, crowd behavior, spectator demographics, and geographic and environmental factors should be taken into consideration during this assessment process. For example, Thackway et al. [41] identified certain characteristics of mass gatherings that require enhanced public health planning and response. These include the duration of the gathering, the number of international visitors, and the geographical extent, which are important factors in determining public health preparedness. While it may not be feasible to implement the same level of effort in all parts of the world, especially in developing countries and contexts with limited resources, such efforts acknowledge the unique aspects and purpose of different mass gathering events, as well as their associated risks and the necessary level of disaster preparedness. Another study [42] examined the perspectives of rescue authorities regarding factors that should be considered in mass gathering preparation. The findings from this study categorized preparedness efforts into the pre-planning phase, factors in the emergency plan, and actions during the event. Taken together, the extent of public health actions should be guided by the outcome of a risk assessment, the availability of resources, and the coordination of responses by relevant agencies [41].

### 4.1. Public health implications

During disasters such as stampedes, it is vital to address the mental health needs of victims and survivors. Our study revealed that tweets expressed negative emotions, including fear, sadness, and anger. These psychological reactions can significantly impact the health and well-being of those affected. Therefore, prioritizing the recruitment and training of professionals who can address the mental health needs of disaster victims and survivors is crucial.

Our findings underscore the importance of preventing disasters during mass gathering events. This aligns with one of the core functions of public health, which involves assessing and monitoring the health of communities and populations at risk to identify health problems and priorities [13]. By contributing to the existing body of literature, which indicates a higher likelihood of stampedes occurring in developing countries, particularly in Asia [6], we emphasize the role of social media as a valuable surveillance tool for advancing disaster science and research. Examining a prevalent health-related hazard associated with sporting events, including the aggravating factors and severity of the damage, has important policy implications. Centralized communication and coordination between the government, local host communities, security agencies, and health agencies during planned mass gatherings is essential. A notable research gap is the limited focus on noncommunicable complications during mass gatherings, with much attention given to infectious diseases (especially during events like COVID-19) [2],[22]. Allocating funding from governments and funding agencies to study the noncommunicable consequences of disasters would provide robust evidence to inform disaster prevention and mitigation policies.

Lastly, our study highlights the increasing role of machine learning and artificial intelligence in predicting and preventing disasters. This underlines the potential for leveraging these technologies in future research and disaster management practices. Embracing these advancements can enhance our disaster preparedness and response capabilities, ultimately leading to more effective and proactive measures.

## 5. Limitation

Our study had several limitations. First, it is important to note that social media users, including Twitter users, may not represent the entire Indonesian population, as individuals of different ages and socio-demographic backgrounds have varying presence on social media and across different platforms [13],[43]. However, despite this limitation, social media users can still reflect a diverse range of demographics and geographic locations, making the information obtained from these platforms relevant for policy and practice.

Second, the prevalence of infodemic, misinformation, and disinformation on social media raises concerns about the validity of the findings from our study. It is crucial to interpret our findings with caution, considering the potential influence of either false or misleading information circulating on social media platforms. Despite these challenges, our study still provides valuable insights into the nature of the disaster and the level of preparedness and response, both in terms of medical and non-medical aspects.

Finally, it is important to acknowledge that our study focused on Twitter comments posted in the English language. This approach may have overlooked perspectives expressed in Bahasa Indonesian, which is the official language of Indonesia. Consequently, our findings may not fully capture the relevant insights that could have emerged from analyzing tweets in the local language, limiting the interpretation of our results.

## 6. Conclusion

This study utilized advanced machine learning techniques to analyze Twitter data and gain insights into public reactions and discourse following a soccer stadium stampede in Indonesia. The findings revealed that the public perceptions and emotions expressed on Twitter were predominantly negative, as characterized by fear, anger, and sadness. A thematic analysis identified various ideas related to the circumstances of the stampede, government responses, and crowd reactions, most of which were associated with negative sentiments. Interestingly, the analysis did not indicate a significant focus on the critical aspect of prehospital medical interventions in the Twitter discourse of the disaster.

From a management perspective, the findings from this study contribute to the existing literature by emphasizing the need for tailored planning and mitigation strategies for different types of events, considering factors such as crowd behavior, demographics, and environmental conditions. In addition, there is a need for comprehensive risk assessments and enhanced public health planning, which are essential for effective disaster preparedness. Additionally, this study underscores the importance of considering mental health interventions for victims and survivors of mass gathering hazards. The negative emotions expressed on Twitter indicate the potential impact on the well-being of individuals exposed to such disasters. Special attention should be given to addressing the mental health needs of affected individuals by recruiting and training professionals in disaster response. Theoretically, our findings add to the existing body of literature by highlighting the higher likelihood of stampedes occurring in developing countries, particularly in Asia. Moreover, this study demonstrates the utility of social media as a surveillance tool for advancing disaster science and research. Central communication and coordination between the government, local host communities, security agencies, and health agencies are essential for effective disaster response.

Future research directions should include a focus on noncommunicable consequences of mass gatherings, as existing studies tend to predominantly address infectious diseases. Allocation of funding and resources to study noncommunicable complications would provide robust evidence to inform disaster prevention and mitigation policies. Additionally, the role of machine learning and artificial intelligence in predicting and preventing disasters should be further explored by researchers.

## Use of AI tools declaration

The authors declare they have not used Artificial Intelligence (AI) tools in the creation of this article.
